# Posttraumatic Atlantoaxial Rotatory Dislocation in a Healthy Adult Patient: A Case Report and Review of the Literature

**DOI:** 10.1155/2012/183581

**Published:** 2012-11-18

**Authors:** Giuseppe Maida, Eleonora Marcati, Silvio Sarubbo

**Affiliations:** Division of Neurosurgery, Department of Neuroscience and Rehabilitation, University-Hospital S. Anna, 8 Via Aldo Moro, Cona, 44124 Ferrara, Italy

## Abstract

Atlantoaxial rotatory dislocation (AARD) is a rare complication in adults usually leading to pain, spinal cord injury, or death. Clinical and radiological diagnosis is difficult and often delayed. We report a rare case of posttraumatic AARD in a neurological intact 27-year-old male in which initial radiographic evaluation was negative. A computed tomography (CT) scan was promptly done because the patient showed a severe torticollis. Therefore, early diagnosis, immobilisation, and surgical fusion and arthrodesis were performed. After surgery, cervical pain and torticollis were resolved and the patient remained neurologically intact with a CT scan documentation of fusion at the 3-year followup.

## 1. Introduction

Craniocervical traumas are commonly described as neurologically catastrophic events with a very low survival rate [1–4]. Moreover, occipitoatlantoaxial dislocations are often misdiagnosed on the initial radiographic and clinical evaluation [5–10]. The delay to diagnosis and treatment has been reported as a negative prognostic factor related to a poor outcome [1, 2, 11]. However, there are a few cases reporting in the literature no neurological deficit in both atlantooccipital and atlantoaxial dislocations [1, 12–15]. Besides, recent improvements in the emergency diagnostic and therapeutic management showed an increased rate of patient survival.

In this paper, we present a rare case of acute posttraumatic atlantoaxial rotatory dislocation (AARD) in a healthy adult patient who underwent early posterior occipito-cervical stabilisation and arthrodesis. 

## 2. Case Report

**Figure 1:**
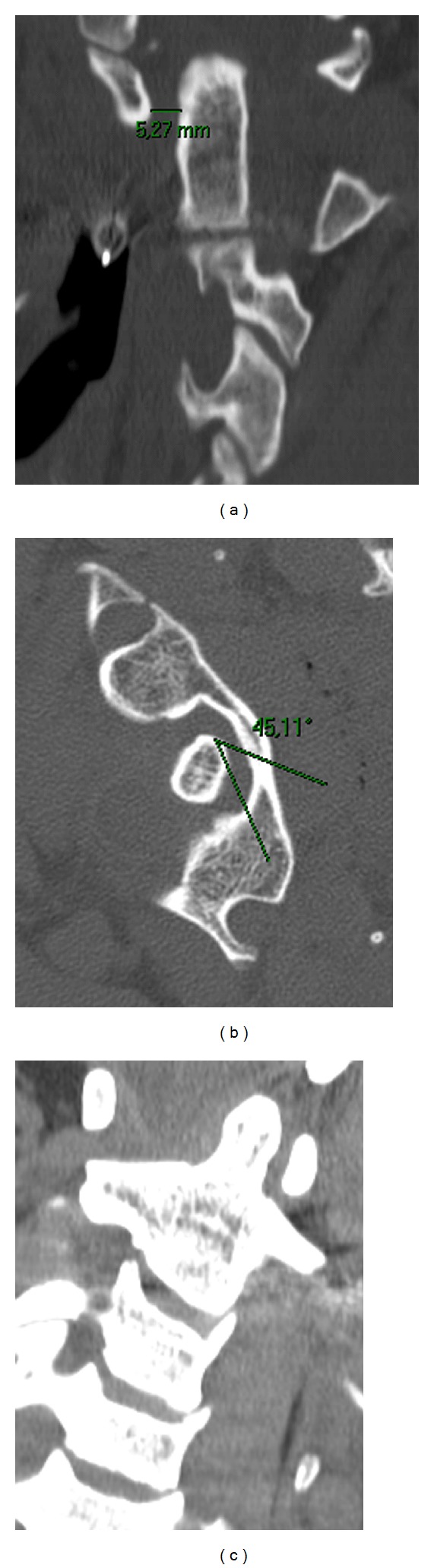
Computed tomography scans showing an atlanto-dental interval greater than 5 mm (a), 45 degrees of rotation (b), and asymmetry of the lateral masses of C1 (c).

**Figure 2:**
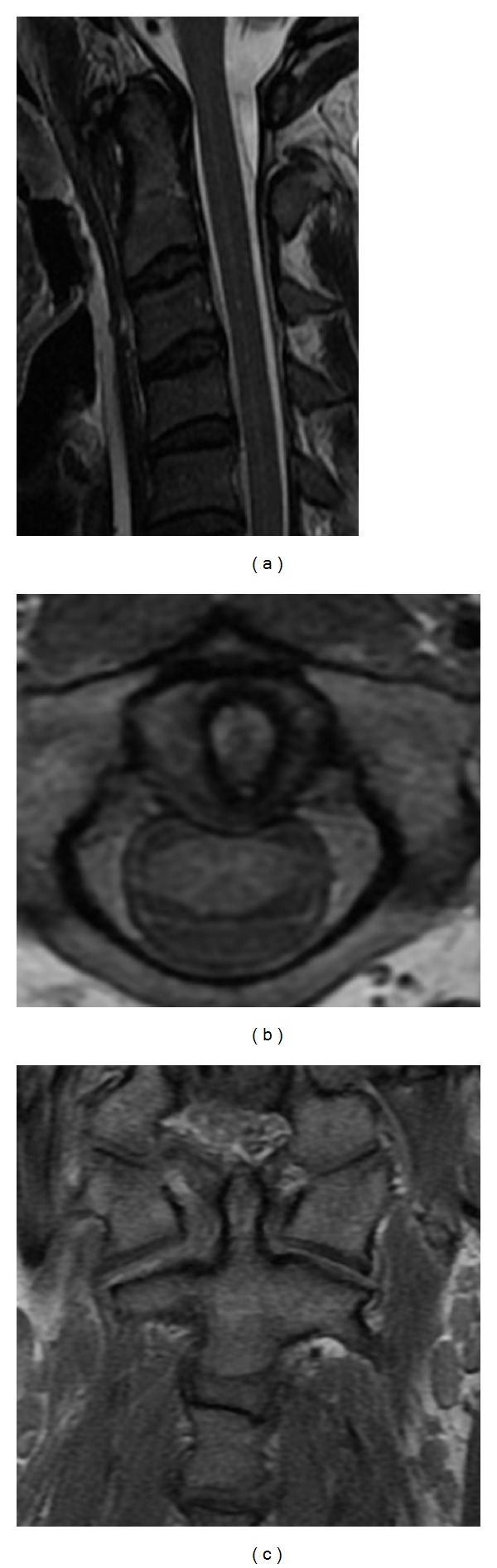
Cervical magnetic resonance imaging showing injury of ligament: midsagittal (a), axial (b), and coronal (c) view.

A twenty-seven-year-old male patient who had been involved in a motor vehicle accident was brought to the emergency room with multiple injuries in October 2008. The patient was not examined on arrival due to the fact that he was intubated shortly after admission for airway protection and hemodynamic instability; however, he was reported to be moving all 4 extremities at the scene. Neutral cervical spine radiographs performed under general anaesthesia revealed no fracture or dislocation. The patient was estubated after a few days and he was found to have a severe torticollis with his head rotated to the left side and tilted toward the right side in a “cock-robin” position, muscles spasms, painful limitation of motion, and inability to turn the head to the right side. However, the neurological examination was normal. At this time, a computed tomography (CT) of the cervical spine with a bone window was performed showing an atlanto-dental interval (ADI) greater than 5 mm (Figure 1(a)), 45 degrees of axial rotation (Figure 1(b)), and no bone fracture. Additionally, an asymmetry of the lateral masses of C1 with respect to the odontoid process was seen on the anteroposterior CT bone scan image and a loss of normal articular apposition at C1-C2 was documented (Figure 1(c)). Furthermore, a magnetic angioresonance was performed to rule out the risk of injury to the vertebral arteries. A manual manipulation was then attempted under general anaesthesia and a quite complete reduction was confirmed with CT bone scan. At this time, a new cervical spine magnetic resonance imaging was performed to determine the disruption of ligaments revealing soft tissues swelling, a Type IA injury of transverse ligament, a complete rupture of the right alar ligament, and a partial injury of the left one (Figures 2(a), 2(b), and 2(c)). No spinal cord compression or signal changes were identified. 

**Figure 3:**
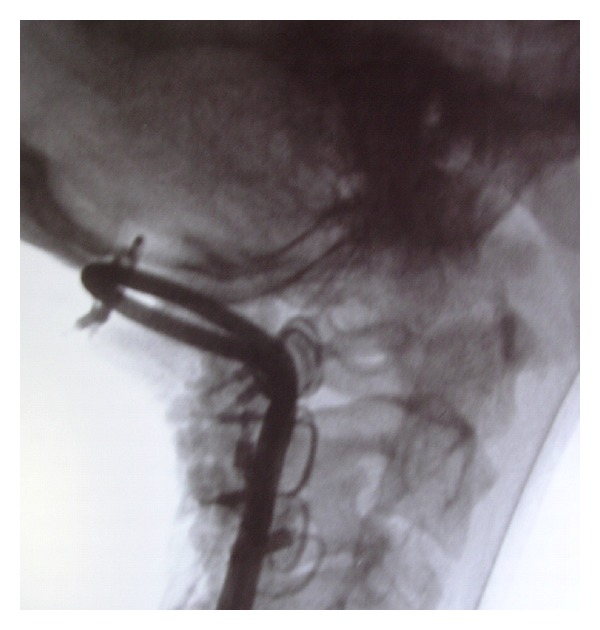
Immediate postoperative radiographs showing the occipitocervical fusion.

**Figure 4:**
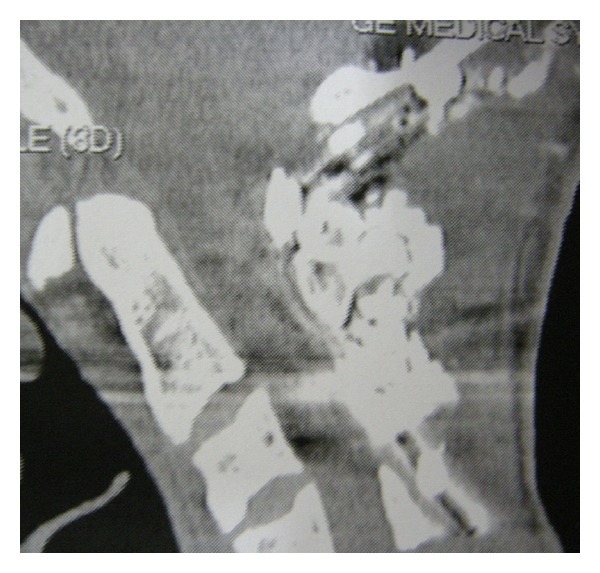
Computed tomography scan showing an occipito-cervical stable fusion at the 3-year followup.

Finally, the patient was placed in a Philadelphia collar and he was transported directly to the operating room. An early posterior stabilisation from the occiput to C3 was performed by using a Hartshill U-shaped rod and Songer sublaminar wires. Moreover, an autologous bone graft, a porcine xenograft, and a biocompatible graft substitute like hydroxyapatite were placed in the decorticated areas. In addition, initial and intraoperative motor and somatosensory evoked potentials suggested no neurological modifications. The postoperative neurological examination did not reveal any deficit and the torticollis was completely resolved. After surgery, the patient was placed in a Philadelphia collar for 60 days. Immediate postoperative radiographs showed a proper implant placement (Figure 3). CT bone scans at 60-day and 3-year followup demonstrated an occipitocervical stable fusion (Figure 4); furthermore, the patient was free of pain and torticollis and returned to his normal daily activities. 

## 3. Discussion 

Craniovertebral junction (CVJ) is a complicated anatomical and functional structure representing the transition between the skull and the spine which allows the extension, flexion, and lateral rotation of the head [16]. In particular, the atlanto-axial segment has singular characteristics when compared with the inferior cervical vertebrae specifically because of its higher range of rotation [5]. 

AARD is a rare pathology of the upper cervical spine in which the physiological replacement to the neutral position after a rotational movement of the head is not allowed [5]. It was described in 1907 by Corner, for the first time [17], and over the past three decades a total of 16 cases were reported in the literature [5, 6, 9, 10, 18–30]. Although the pathogenesis is still not well defined, two major hypotheses are suggested in this regard: the interposition of synovia as a mechanical obstacle and the partial or total injury of the main ligamentous structures that normally limit the excessive anterior shift and rotation (the transverse and the alar ligaments, resp.) [6, 10, 31, 32]. Generally, traumatic events or infections may cause the AARD. Nevertheless other conditions can be also associated, such as inflammation of the nasopharynx, postoperative procedures involving the head and the neck, congenital laxity of ligaments (i.e., Down's syndrome, Marfan's syndrome, or rheumatoid arthritis), metastatic tumors, and eosinophilic granulomas [1, 10, 33–39]. Posttraumatic AARD is very rare in adults probably because it is often related to lethal injuries due to a severe associated trauma [10, 18, 19, 24, 35, 40–42]. However, many authors suggest that the higher incidence of AARD in children may be dependent to specific anatomical features, such as a more horizontal and shallow joint surface of the lateral mass, a relative elasticity of the ligaments, not-fully developed neck muscles, and a relatively large head [7, 10, 22, 28, 43, 44]. Fielding and Hawkins described 4 types of AARD (Table 1) [6]. This classification, which has been widely accepted, correlates with an increasing risk of spinal instability and potential neurological impairment [35]. This case was categorized as Type III for both the increase of ADI and the injury of ligaments.

Moreover, Dickman et al. have classified the injuries involving the transverse atlantal ligament or its osseous insertions (Table 2). In this classification, the disruption of the ligamentous substance was named as Type I and fractures or avulsions involving the tubercle which affords insertion of the transverse ligament on the C1 lateral mass were named as Type II. Type I injuries, as in our case, are considered incapable of healing without internal fixation and they should be treated with early surgery [45].

Accordingly, in our case, we decided to perform an early internal fixation extended from occiput to C3. In fact, they were documented either the injury of the transverse ligament and the injury of alar ligaments thus compromising the stability of C1-C2 and C0-C1, respectively [1–3, 5, 45]. In addition, the 45 degrees of atlanto-axial rotator dislocation and the increase of the ADI to 4 mm met the C0-C2 instability criteria as described by White and Panjabi [44]. In these cases, an occipito-cervical fusion procedure is generally considered as neuroprotective and should be performed as early as possible [1, 46–49]. 

Numerous surgical techniques were proposed for the occipito-cervical fusion [50–57]. Until now, there is no Class I evidence about the use of wires or screw. The wiring/rod method is probably one of the simplest, least expensive, and most widely available worldwide. This method allows the reduction of the dislocation and provides immediate rigid fixation [57]. 

Although immediate fixation is achieved, long-term stability can be improved by bone arthrodesis and we have thus included grafting [58–60]. 

## 4. Conclusion

The diagnosis of AARD should be suspected each time torticollis and cervical pain are associated. However, the AARD can also occur in the absence of any clinical sign and thus it should be systematically considered and excluded in any traumatic patient. The radiographic findings on the usual anteroposterior and lateral radiographs of the cervical spine may be difficult to interpret because of the rotatory deformity. Therefore, the CT scan is to be considered the gold standard and it must be promptly provided. Moreover, magnetic resonance imaging is also mandatory to rule out neurovascular injuries and to identify eventual ligamentous ruptures. When instability of the craniovertebral junction is diagnosed, early reduction and fusion are the recommended definitive treatments to prevent neurologic deterioration and vertebral arteries injury. Although numerous surgical techniques have been developed, to achieve a successful outcome surgeons should be familiar with the atlanto-axial complex and the selected method. We chose the wiring/rod method which is simpler, less expensive and provides immediate semirigid fixation and excellent fusion results. 

## Figures and Tables

**Table 1:** Classification of AARD by Fielding and Hawkins [6].

Type	Injury of transverse ligament	ADI
Type I	None	<3 mm
Type II	Mild	3–5 mm
Type III	Deficiency both of the transverse and alar ligaments	>5 mm
Type IV	Deficiency both of the transverse and alar ligaments	Posterior shift of the atlas

*Adapted by Kim et al., 2007 [34].

**Table 2:** Classification of transverse ligament injuries by Dickman et al. [45].

Type	Injury of transverse ligament
Type I	Disruption of ligament substance
	(A) Midportion
	(B) Periosteal insertion
Type II	Disconnection of the tubercle of insertion from the C1 lateral mass
	(A) Comminuted fracture of C1 lateral mass
	(B) Avulsion with an intact C1 lateral mass
